# National Institutes of Health Stroke Scale (NIHSS) scoring inconsistencies between neurologists and emergency room nurses

**DOI:** 10.3389/fneur.2022.1093392

**Published:** 2023-01-11

**Authors:** Amber R. Comer, Evan Templeton, Michelle Glidden, Stephanie Bartlett, Lynn D'Cruz, Donya Nemati, Samantha Zabel, James E. Slaven

**Affiliations:** ^1^Indiana University School of Health and Human Sciences, Indianapolis, IN, United States; ^2^Indiana University School of Medicine, Indianapolis, IN, United States; ^3^Eskenazi Health, Indianapolis, IN, United States

**Keywords:** stroke, NIHSS scoring, inconsistency, neurologists, nurses

## Abstract

**Background:**

Little is known about the consistency of initial NIHSS scores between neurologists and RNs in clinical practice.

**Methods:**

A cohort study of patients with a code stroke was conducted at an urban academic Primary Stroke Center in the Midwest between January 1, 2018, and December 31, 2019 to determine consistency in National Institutes of Health Stroke Scale Scores (NIHSS) between neurologists and registered nurses (RNs).

**Results:**

Among the 438 patients included in this study 65.3% (*n* = 286) of neurologist-RN NIHSS scoring pairs had congruent scores. One-in-three, (34.7%, *n* = 152) of neurologist-RN NIHSS scoring pairs had a clinically meaningful scoring difference of two points or greater. Higher NIHSS (*p* ≤ 0.01) and aphasia (*p* ≤ 0.01) were each associated with incongruent scoring between neurologist and emergency room RN pairs.

**Conclusions:**

One-in-three initial NIHSS assessed by both a neurologist and RN had a clinically meaningful score difference between providers. More severe stroke, as indicated by a higher NIHSS was associated with scoring inconsistency between neurologist-RN pairs. Subjective scoring measures, especially those involving a patient having aphasia, was associated with greater score incongruency. Score differences may be attributed to differences in NIHSS training requirements between neurologists and RNs.

## Introduction

Initially designed for use in clinical research trials, the National Institute of Health Stroke Scale Score (NIHSS) has become the gold standard for bedside clinical stroke assessment (1). This valid and reliable 15-item scale captures baseline deficits to determine stroke severity and symptom intensity (2, 3). Additionally, serial use of the NIHSS has been shown to track changes in stroke severity due to recanalization, worsening clot proliferation or resolution of diaschisis and early plasticity (4). As the NIHSS contains items that have the capacity to be subjectively interpreted, effective and consistent NIHSS scoring requires training (5, 6). The NIHSS is the most widely used stroke severity rating scale in neurology with over 500,000 practitioners trained through the NIH in its administration (1).

In many cases, neurologists learn to utilize the NIHSS during their residency while working in the hospital. Neurologists can obtain certification in NIHSS scoring, which is recommended as part of primary stroke center (PSC) certification by the Joint Commission; however, this training is not mandatory (7). While NIHSS scoring training is not mandatory for neurologists, Registered Nurses (RNs) working in emergency departments, trauma/intensive care units, and neurology units are required to train and certify through the NIH annually for hospitals to retain PSC designation. While neurologists and RNs perform the NIHSS separately, consistency in scoring indicates accuracy in patient assessment and guides further testing, imaging, and treatment (3, 8).

Although it is common in clinical practice for multi-raters to administer NIHSS scores, the only known study comparing scores between provider specialties was collected during a clinical research trial and showed better interrater reliability among RNs (9). Little is known about the consistency of initial NIHSS scores between neurologists and RNs in clinical practice. This study sought to evaluate the congruency between neurologists and RN initial NIHSS scores for stroke patients and to determine patient and clinical factors that may influence NIHSS scoring inconsistencies.

## Methods

A retrospective chart review was performed on all code stroke calls at a 315-bed urban academic PSC in the Midwest between January 1, 2018, and December 31, 2019. A total of 588 patients, over the age of 18, in which a code stroke was activated were identified by the hospital's stroke coordinator for inclusion in this study. Patients with hemorrhagic stroke (*n* = 38) or with missing NIHSS initial scores from either the neurologist or RN (*n* = 77) were excluded from this study. Additionally, patients with more than a 60-min time difference between initial neurologist and RN NIHSS administration (*n* = 35) were excluded because it was determined that a >60-min time frame between scores may alter NIHSS scoring due to stroke progression or symptom resolution. All RNs in this study were NIHSS-certified through the National Institute of Health; however, not all neurologists were NIHSS certified. In this center, the initial NIHSS is scored by the neurologist; however, for monitoring purposes, the RN conducts an NIHSS screening within 30 min of the initial response. The university IRB approved this study.

### Interrater agreement

Before data collection, the research team created a written data collection instrument that described individual variables. Chart reviewers used ten charts for interrater agreement of each variable using the Shrout and Fleiss method for fixed effect and average measure of agreement (10). The calculated kappa statistic for each variable collected ranged from 0.8980 to 1.0, indicating a good to excellent level of agreement among the data-collecting researchers (10).

### Data collection

Initial NIHSS scores as recorded by neurologists and RNs were the primary outcome measures of this study. Initial NIHSS scores and timestamps were obtained from neurology and RN notes. Demographic information was collected on all patients. Patient and clinical characteristics collected from medical charts consisted of administration of tissue plasminogen activator (tPA), tPA administration timestamp, and whether thrombectomy was performed. Additionally, discharge diagnosis, aphasia, previous cerebrovascular accident (CVA), or transient ischemic attacks (TIA), and multiple comorbidities, including myocardial infarction (MI) and coronary artery disease (CAD), were collected.

### Data analysis

Two cohorts were created based on the point difference between the neurologist and RN NIHSS scores. Cohort 1 consisted of neurologist-RN NIHSS scoring pairs with a score difference of less than two points. Cohort 2 consisted of neurologist-RN scoring pairs with a clinically meaningful score difference of two points or greater (2, 4). The 2-point difference was selected as meaningful for several reasons. First and most importantly, the score differential of 2 has been used to denote a meaningful difference in other studies which assess score differences between providers (emergency medicine vs. neurology) and is used consistently throughout the literature (2, 11). Second, this point difference can change the stratification of stroke severity. For example, a patient scored as an NIHSS of 4 by an RN would be classified as a minor stroke; however, that same patient with an NIHSS of 6 scored by a neurologist would be classified as a moderate stroke. Furthermore, the NIHSS is intended to be a standardized score, and therefore, there should not be major delineations between scorers.

Demographic data of all participants was analyzed using descriptive statistics. Univariate comparisons using chi-square were performed to assess differences in NIHSS scores. The Fisher's exact test was performed for low-frequency dichotomous variables (*n* ≤ 5). The Wilcoxon rank-sum test was conducted to evaluate differences in categorical and continuous variables as appropriate. Generalized linear regression was performed to determine patients' demographics and clinical characteristics associated with NIHSS scores discrepancy for Cohort B. All analyses were performed using SAS 9.4 (SAS Institute, Cary, NC), and *p*-value was set at *p* < 0.05 (two-tailed).

## Results

A total of 438 patients were included in data analysis. A total of *n* = 286 (65.3%) neurologist-RN NIHSS scoring pairs were less than two points difference and were placed in Cohort 1. A total of *n* = 152 (34.7%) of neurologist-RN NIHSS scoring pairs had a clinically meaningful scoring difference of two points or greater and were placed in Cohort 2. The mean age of all patients was 55.5 ± 13.8 years (range 21–101); 51.8% were men; 44.6% were Caucasian, and 43.3% were African American (Table 1). Cohorts did not vary in demographics including age, sex, and race (Table 1). No association was found between demographic and clinical characteristics and score inconsistency.

**Table 1:** Demographics and clinical characteristics of total group and cohorts.

**Variables**	**All subjects**,	**Cohort 1**	**Cohort 2**	**Chi Square**
	***N* = 438**	***n* = 286**	***n* = 152**	***p*-value**
		**(65.3%)**	**(34.7%)**	
**Demographics**				
Age (years), mean (SD)	55.5	55.2	56.1	0.501
Range	(13.8); 21–101	(14.1); 21–101	(13.2); 21–93	
**Age group**				
(< 65)	334 (76.3)	218 (65.3)	116 (34.7)	0.9828
65+	104 (23.7)	68 (65.4)	36 (34.6)	
**Sex**				
Female	209 (48.2)	142 (67.9)	67 (32.1)	0.2903
Male	225 (51.8)	142 (63.1)	83 (36.9)	
**Race**				
Caucasian	195 (44.6)	127 (65.1)	68 (34.9)	0.3906
African American	189 (43.3)	120 (63.5)	69 (36.5)	
Others	53 (12.1)	39 (73.6)	14 (26.4)	
**Medical history**				
Aphasia	222 (50.7)	129 (58.1)	93 (41.9)	0.0014
Atrial fib	34 (7.8)	21 (61.8)	13 (38.2)	0.6383
Carotid Stenosis	11 (2.5)	8 (72.7)	3 (27.3)	0.7546^†^
CAD/MI	100 (22.8)	64 (64.0)	36 (36.0)	0.7565
TIA	40 (9.2)	28 (70.0)	12 (30.0)	0.5052
Dementia	18 (4.1)	10 (55.6)	8 (44.4)	0.3753
Diabetes	180 (41.3)	117 (65.0)	63 (35.0)	0.8926
Hypertension	333 (76.0)	219 (65.8)	114 (34.2)	0.7135
PVD	9 (2.1)	6 (66.7)	3 (33.3)	>0.9999^†^
Past CVA	187 (42.9)	115 (61.5)	72 (38.5)	0.1669
**Baseline NIHSS**				
Neurologist-RN paired difference, median (IQR)	1 (0, 2)	0 (0, 1)	3 (2, 4)	< 0.000^‡^
Range	0–13	0–1	2–13	
**Treatments**				
tPA	78 (17.8)	55 (70.5)	23 (29.5)	0.2858
Thrombectomy	25 (5.7)	17 (68.0)	8 (32.0)	0.07700
**Stroke diagnosis**				
No stroke	207 (47.5)	135 (65.2)	72 (35.8)	0.7320
Ischemic stroke	191 (43.8)	123 (64.4)	68 (35.6)	
TIA	38 (8.7)	27 (71.1)	11 (29.0)	

Cohort 1 = score difference < 2, Cohort 2 = score difference ≥ 2; Values are means (standard deviations) for age and frequencies (percentages) for categorical variables, with p-values from Student's t-test and Chi-Square (verified with Fisher's Exact) tests.

† Due to low count in some cells used the Fisher's Exact Test.

‡ Between group differences at p ≤ 0.05; Wilcoxon Rank-Sum Test.

Among all patients, more than half (50.7%) presented with aphasia, of which 58% (129) patients did not have a meaningful score difference between neurologists and RNs (*p* ≤ 0.01). Distribution was non-significant between cohorts for receipt of tPA, thrombectomy, and discharge diagnosis (*p* = 0.05). The median NIHSS scores were significantly different between the two cohorts, with Cohort 2 having higher NIHSS scores (*p* ≤ 0.01) (Table 1).

## Discussion

This is among one of the first studies comparing NIHSS scoring between neurologists and RNs during clinical practice. This study found that NIHSS scores were consistent between neurologists and RNs in two-thirds of patients, supporting previous reliability studies (6, 9, 12). However, one-third of patients (34.7%) had a clinically meaningful score difference of two points or greater between neurologist and RN pair initial NIHSS. This discrepancy in NIHSS scores may lead to treatment approaches that are overly conservative or aggressive for stroke (9).

Higher NIHSS was associated with Neurologist-RN pairs having a clinically meaningful score difference between providers. This suggests that stroke severity may account for inconsistent NIHSS scoring between neurologists and RNs. Patients presenting with aphasia were more likely to have incongruent scores between neurologists and RNs as 60% of patients with aphasia had an incongruent score when compared to patients without aphasia (40% incongruent scores). The incongruence between neurologist-RN pairs when scoring aphasic patients likely exists because this portion of the NIHSS exam relies on the patient following instructions and to respond to things that are not straight forward such as a sensory examination. For example, scoring differences may be attributed to the contribution of neurological clinical skills in the examination of patients with severe stroke and/or aphasia, hemianopia or hemispatial neglect. Neurologists may not follow the NIHSS recommended guidelines for aphasia classification and may in essence give patients “credit” while scoring based on what they believe the patient may be capable of and not necessarily score what they see the patient is doing or not doing in the moment. It is suspected that well-meaning providers may give the patient the benefit of the doubt and score what they think the patient is capable of rather than scoring what they actually see. Conversely, congruent pairs were more likely to have lower NIHSS scores. This is likely because the other areas of the NIHSS are objective. The objective portions of the NIHSS lead to more consistent scoring than measures that require a degree of subjective interpretation such as interpreting the abilities of patients with aphasia. The findings of this study show the importance of the NIHSS adage, “score what you see.”

Conversely, it is possible that the scoring differences are related to a misinterpretation of the patient's ability to understand the scoring cards. For example, studies have found cultural differences the language used to describe pictures on the scoring cards. Whereas, in the United States the concept of a hammock is a well-known concept, other cultures may not know what a hammock is and may respond with “I do not know” which would result in a score delineation between scorers if one scorer accepts this answer due to cultural differences and another scorer does not. This is an important concept for the patient population within this study as the medical center in which study was conducted as a high prevalence of English as a second language patients. Future studies should be conducted to evaluate whether discordance in clinical training is indeed responsible for NIHSS scoring differences (13, 14).

This study has several limitations. First, the study was retrospective, thus we were unable to ascertain providers scoring rationale. Second, this study was conducted at a single hospital and may not be generalizable. There are two reasons why scores may not match: (1) the scoring is inconsistent; or (2) the patient is experiencing change. In order to control for the patient experiencing change, we excluded neurologist-RN NIHSS scoring pairs if one or both of the scores occurred after the patient received thrombolysis. Additionally, we limited the time frame between neurologist-RN pairs to a 60-min window in order to minimize scoring differences resulting from a change in the patient's clinical status. We acknowledge that large strokes are dynamic events and therefore, it is possible that the scoring differences were related to the patient changing and necessarily a scoring difference. Lastly, given the study design, this study was unable to determine the experience of the nurses and neurologists. Future research to determine differences in provider experiences when conducting an NIHSS evaluation is needed.

This study found that 1-in-3 neurologist-RN initial NIHSS scores had a clinically meaningful score difference and that differences were more common in patients with higher NIHSS and aphasia. Clinical features can make performing the NIHSS difficult, especially for inexperienced clinicians, education about how to standardize the scoring of these specific areas of the NIHSS scoring system that have room for interpretation may improve concordance of scoring between providers. It is possible that inconsistent scoring resulted from differences in NIHSS training requirements for neurologists and RNs; however, further research is needed to determine if changing training requirements would improve scoring incongruencies.

## Data availability statement

The raw data supporting the conclusions of this article will be made available by the authors, without undue reservation.

## Ethics statement

The studies involving human participants were reviewed and approved by IUPUI IRB. Written informed consent for participation was not required for this study in accordance with the national legislation and the institutional requirements.

## Author contributions

Authors contributed to all aspects of the research project, from the design, interpretation, and editing: AC, ET, MR, SB, LD'C, DN, SZ, and JS. Implementation: AC, ET, MR, SB, LD'C, DN, and SZ. Data collection and first draft: SB, LD'C, DN, and SZ. Analysis: AC and JS. Last revision of the final manuscript: AC and ET. All authors approved the manuscript for publication and agree to account for all aspects of the work.
